# Assessing the Influence of Child Sexual Behavior on Depression among Black SMM in the Southeastern United States

**DOI:** 10.3390/ijerph192113930

**Published:** 2022-10-26

**Authors:** Donte Travon Boyd, Gamji Rabiu Abu-Ba’are, Ashleigh LoVette, Darren L. Whitfield, Rodman E. Turpin, S. Raquel Ramos, Camille R. Quinn, DeMarc A. Hickson

**Affiliations:** 1 College of Social Work, Ohio State University, Columbus, OH 43210, USA; 2 Us Helping Us, People into Living, Inc., Washington, DC 20010, USA; 3 Department of Public Health Sciences, University of Rochester, Rochester, NY 14642, USA; 4 School of Nursing, University of Rochester, Rochester, NY 14642, USA; 5 Center for Interdisciplinary Research on AIDS, New Haven, CT 06511, USA; 6 College of Public Health, Ohio State University, Columbus, OH 43210, USA; 7 School of Social Work, University of Maryland, Baltimore, MD 21201, USA; 8 Department of Global & Community Health, College of Health and Human Services, George Mason University, College Park, MD 20742, USA; 9 School of Nursing, Yale University, West Haven, CT 06477, USA; 10 Center for Equitable Family and Community Well-Being, School of Social Work, University of Michigan, Ann Arbor, MI 48109, USA

**Keywords:** SMM, depression, US south, child sexual abuse, childhood maltreatment

## Abstract

Limited studies have examined the associations between child sexual abuse (CSA) and depression among Black sexual minority men (SMM) in the Southeastern United States (US). As, such, the current study examined the critical gap in understanding the impact of CSA on Black SMM’s mental health. Specifically, we tested the associations between contextual CSA factors and depression among a large population-based sample of Black SMM living in two cities in the Southern US. Data were obtained from the MARI Study, a sample of Black SMM ages 18–66 years, recruited from the Jackson, MS and Atlanta, GA metropolitan areas (*n* = 507). Depression was assessed using the 9-item CES-D scale. We conducted multivariable regression analyses to examine the association between depression with history of CSA and other child sexual-related variables (i.e., age of perpetrator and age of sexual abuse), controlling for key confounders. Our results indicated that CSA (β = 0.14, *p* < 0.001) was positively associated with depression. Our results also indicated that Black SMM who reported being sexually abused at the ages of 6 to 10 (β = 0.30, *p* < 0.01) and 16 to 18 (β = 0.25, *p* < 0.05) were positively associated with depression. These findings suggest that there is a need to provide culturally and safe mental health services in the Southeastern US for CSA survivors.

## 1. Introduction

Adverse mental health outcomes, such as those linked to depression, can have a widespread impact on the lives and well-being of individuals and society [1,2]. According to prior research, depression is common among sexual minority men (SMM) [3]. For example, one study found that SMM have a three times greater chance of a major depression diagnosis than heterosexual men [3,4]. Additionally, the meta-analyses from a systematic review found that over the course of 12 months, depression and anxiety disorders occurred over 1.5 times more in lesbians, gays, and bisexuals compared to non-LGBTQ+-identified persons [5]. Depression, a common yet stigmatized mental health condition, is defined as a negative affective state. It comprises more than feelings of sadness and frequently coexists with other mental health issues such as substance use. If left untreated, it can have dire health consequences for sexual minorities [6,7,8].

### 1.1. US South and Mental Health

The dual burden of both sexual minority stigma and racial discrimination can aggravate health stressors among Black SMM. Specifically, highlighting the interconnectedness of these two experiences illustrates a critical need to consider the impact of the marginalization associated with intersecting identities when studying mental health disparities [9,10,11]. For instance, Black SMM have higher negative mental health outcomes originating from the stigma of identifying as sexual minorities [3]. Black SMM also encounter numerous challenges in accessing mental health services and support due to existing race-based disparities in health care access, further disadvantaging them [12,13]. These disparities are compounded and stem from a wide range of factors, including historical trauma, structural racism, institutional policies of mass incarceration and substance misuse, and individual-level challenges [14,15,16,17,18]. Moreover, these issues are particularly salient in the Southern United States (US).

According to 2010 census data, more than half (55%) of the Black US population resides in the US South, and research focusing on this demographic area should be a priority concern [19,20]. When examining differences in access to mental health resources, accounting for environmental and cultural factors is especially important for Black men, as studies have shown that these factors uniquely influence help-seeking behaviors [21,22,23]. Research on mental health, especially factors associated with depression among Black SMM in the US, remains limited [24,25,26,27]. For instance, Wade and Harper [28] revealed that only 18.5% of articles on Black SMM included content on depression and indicated an overall lack of research addressing the psychosocial functioning among them [28]. However, the few studies that focus on Black SMM indicate the high prevalence of depression and mental health concerns among Black SMM [28,29]. In a study among urban young SMM, Burns [29] estimated a 33.2% prevalence of lifetime major depressive episodes among Black SMM.

### 1.2. CSA and Black SMM

While numerous factors contribute to adverse mental health outcomes, childhood traumatic experiences such as CSA frequently relate to depression (i.e., psychopathology) in adulthood [30,31,32]. CSA has varying definitions, but it often refers to unwanted or forced sexual contact before the age of 17 and/or sexual contact with someone four to five years older at any time during childhood [33,34]. CSA is nearly five times greater among SMM in comparison to the general men population [35]. Studies focusing on racial and sexual minorities have indicated a high prevalence of history of CSA among Black SMM [35,36]. A study focused on Black SMM in New York City reported a prevalence of 28.1% for CSA [37]. Another multisite study across six cities, including one Southern US city (Atlanta), identified high rates of both CSA (54%), among Black SMM [38].

Despite the intersectional existence of depression and CSA among Black SMM, limited research exists on the association between the CSA and depression among Black SMM. Recently, more attention has been paid to the need to address the limited availability of research examining how CSA relates to mental health among racial and sexual minorities, especially in the Southern US [39,40]. Understanding how CSA influences mental health outcomes (e.g., depression) will help the development of policies and interventions addressing mental health disparities experienced by Black SMM, especially those in poorly resourced areas such as the Southern US. The limited studies examining CSA and depression suggest that CSA may be associated with adulthood mental stress and depression and associated issues such as risky sexual behaviors and substance use among Black SMM [38,41]. A formative sexual experience interview revealed a high prevalence of CSA among Black SMM, and that unnoticed CSA among Black SMM resulted in unhealthy behaviors characterized by depression and other mental stressors (e.g., substance abuse and high-risk sexual behaviors) [41]. Similarly, Williams et al. [38] found high depression and likelihood of multiple sexual partnership among a large sample of Black MSM who experienced CSA. 

### 1.3. Current Study

A dearth of literature that examined childhood trauma and depression among Black SMM especially in the US South focused on the relationships between depression and CSA among Black SMM, primarily because of associations of CSA with increased HIV and sexual risk among Black SMM in adulthood [42,43,44]. Moreover, previous research in this area has primarily used qualitative methods to explore mental health and well-being related to abuse and trauma among racial and sexual minority populations [40,41].

In this paper, we add to the existing literature and broaden the scope of knowledge by quantitatively examining critical gaps in understanding of the impact of CSA on Black SMM’s mental health. Specifically, we tested the associations between contextual CSA factors and depression among a large population-based sample of Black SMM living in two cities in the Southern US. The study also included the prominent role of disclosure of sexual abuse, as limited information exists on the relationship between childhood trauma, disclosure, and depression among racial and sexual minorities, such as Black SMM in the U.S South.

## 2. Methods

For this cross-sectional study, we used data from the Ecological Study of Sexual Behaviors and HIV/STI among Black/African American SMM in the Southeastern US (known locally as The MARI Study). The MARI Study is a two-city, population-based study designed to typify the HIV environmental “riskscape” and to identify the risk and protective factors of sexual behaviors and STIs among Black SMM in Jackson, Mississippi, (Copiah, Hinds, Madison, Rankin, and Simpson counties) and Atlanta, Georgia (Clayton, Cobb, DeKalb, Fulton, and Gwinnett counties). Participants were recruited through: (1) distribution and posting of printed advertisements at local colleges and universities, adult bookstores, bars, and clubs, as well as community-based organizations (CBOs) that provide healthcare and social services to Black SMM; (2) face-to-face recruitment from local bars and clubs frequented by Black SMM, as well as from HIV prevention interventions, community events, and other activities conducted by local CBOs; (3) social networking websites and apps such as Facebook; (4) geospatial sexual networking apps such as Jack’d; and (5) word-of-mouth referrals from participants and CBO staff not affiliated with the study. Eligibility included the following criteria: (1) self-identification as African American or Black; (2) assigned male at birth (cis males and transgender women); (3) aged 18 years or older; (4) residence in the Jackson, MS, or Atlanta, GA, metropolitan statistical areas; and (5) reported oral or anal sex with another man in the past six months. The total sample for this study was 568 SMM residing in the Southeastern United States with valid survey and physiological data. The study protocol was approved by the Sterling Institutional Review Board and all participants provided signed informed consent.

## 3. Study Measures

### 3.1. Depression

Participants completed the Center for Epidemiological Studies Depression Scale (CES-D) to assess current depressive symptomatology [45]. They rated the frequency that they experienced symptoms over the past week (e.g., “I felt that I could not shake off the blues even with help from my family or friends.”). The response frequencies for the 20 symptoms were rated on a scale from 0 (0 days) to 3 (5–7 days). Items were then averaged into a composite score [46,47]. The Cronbach alpha was 0.91.

### 3.2. Childhood Sexual Abuse (CSA)

Several variables were collected to capture the CSA experiences of Black SMM. Childhood sexual abuse was defined as an experience before the age of 18 where the participant was pressured, forced, or intimidated into doing something sexual that they felt uncomfortable with or did not want to do [38,48]. CSA was based on a 6-item, dichotomous (Yes/No) response asking the participant questions such as “someone touched or fondled your sex organs” and “another person performed oral sex on you”. Internal reliability of the scale was good for this sample (α = 0.90). Age when the abuse happened was measured using a single item, 5-point Likert scale type question ranging from (1 = 1 to 5 years to 5 = Never Happened). Age of perpetrator was estimated using a single item, 5-point Likert scale type question ranging from (1 = 13 to 18 years old to 5 = Never Happened). The sex of the perpetrator was measured using a single item, with a dichotomous response (0 = male, 1 = female). Bothered by CSA was measured using a single item, 4-point Likert type question “How much did the experience bother you at the time?” with responses ranging from (1 = a little to 4 = a lot).

### 3.3. Sociodemographic Measures

Several contextual variables were collected. Age (1 = 55 and above, 2 = 35 to 54, 3 = 25 to 34, 4 = 16 to 24) was treated as a categorical variable in this study. Participants self-reported their HIV status (1 = HIV-negative, 2 = HIV-positive, or 3 = unknown). Employment status was measured using a single item, 4-point Likert type scale question ranging from 1 = Working now, full-time to 4 = Unemployed not looking for work. Education was measured as the highest level of education attained from six options: less than high school; high school diploma or GED; some college, trade, or vocational school; college graduate; graduate school; more than graduate school. Childhood family structure was measured using a single item, 6-point Likert type scale question ranging from 1 = two-parent household to 6 = guardian.

## 4. Analysis

All analyses were conducted on observations that included non-missing data for the outcome variable CSA. Statistical tests of association were conducted between the predictors and the outcome. Bivariate regression models were conducted between the independent variables (CSA, age of abuse, age of perpetrator, the sex of the perpetrator, and currently bothered by CSA) and the dependent variable, depression. Next, multivariable regression was conducted with predictor and sociodemographic variables on the outcome variable depression. Survey data were analyzed based on listwise deletion. For survey scales, a mean score of the scale items was generated for participants with non-missing data. All analyses were conducted in STATA 17.

## 5. Results

### 5.1. Participant Characteristics

Table 1 presents sociodemographic characteristics of Black SMM in the US South. The mean age was 33.6 (*SD* = 12.8, range: 17–68) years old and most of the sample had a mean household income below the federal poverty line. Over a third (40%) attended some college or had obtained some college, trade, or vocational school. Approximately 40% were unemployed but looking for work. More than half (58%) of the sample self-reported renting a place to live.

More than half (53%) grew up in a single-mother household. More than half (53%) of the sample reported CSA, and almost 75% of the Black SMM reported still being bothered by CSA (see Table 2). The majority stated the perpetrator was a male (90%) and between the ages 16 to 24 (40%). Twenty three percent of the sample self-reported they were sexual abused between the ages 11 to 18. Approximately 42% of Black SMM reported experiencing depression (Table 2).

### 5.2. Bivariate Regression

The results of the unadjusted bivariate correlations are presented in Table 3. Our results indicate that sexual abuse was statistically significant and positively and marginally associated with depression (β = 0.13, *p* < 0.10). Individuals who reported experiencing sexual abuse between the ages of 16 to 18 were more likely to experience depression symptoms (β = 0.14, *p* < 0.10). Black SMM who reported experiencing CSA by a perpetrator between the ages of 31 and older (β = 0.10, *p* < 0.05) were statistically significant and positively associated with depression. The sex of the perpetrator, both male (*β* = 0.22, *p* < 0.001) and female (β = 0.22, *p* < 0.001), were both statistically significant and positively associated with depression. Black males who grew up in single mother (β = 0.14, *p* < 0.01), single father (β = 0.09, *p* < 0.05), or stepparent households (β = 0.11, *p* < 0.05) were more likely to report depression than those in two-parent households. Being HIV positive was statistically significant and associated with depression (β = −0.10, *p* < 0.01). Age groups 16 to 24 (β = 0.54, *p* < 0.001), and 25 to 34 (β = 0.39, *p* < 0.001) were less likely to report depression in comparison to adults 55 and older. Being unemployed and looking for work was statistically significant and negatively associated with depression (β = −0.11, *p* < 0.05). Those unemployed and not looking for work were statistically significant and positively associated with depression symptoms (β = 0.07, *p* < 0.05).

### 5.3. Multivariate Regression

The results of the adjusted multiple regression are presented in Table 4. Our results indicate that childhood sexual abuse was statistically significant and positively associated with depression (*β* = 0.14, *p* < 0.01) while holding all other variables were constant. Black SMM who self-reported they were sexually abused at the ages of 1 to 5 (*β* = 0.29, *p* < 0.05), 6 to 10 (*β* = 0.30, *p* < 0.05), 16 to 18 (*β* = 0.25, *p* < 0.01), and do not know (*β* = 0.15, *p* < 0.05) were more likely to report depression symptoms than those who stated that it never happened. The sex of the perpetrator, both male (*β* = 0.22, *p* < 0.001) and female (*β* = 0.19, *p* < 0.001), were statistically significant and associated with depression symptoms. Individuals who reported not being bothered by their experience of CSA were statistically significant and negatively associated with depression symptoms (*β* = −0.19, *p* < 0.05). Black SMM grew up in a single mother (*β* = 0.10, *p* < 0.05), single father (*β* = 0.10, *p* < 0.05), or stepparent households (*β* = 0.10, *p* < 0.05) were more likely to report depression symptoms than those in a two-parent household. 

## 6. Discussion

The purpose of this paper was to examine the relationship between depression and history of CSA among Black SMM, including the prominent role of disclosure of sexual abuse. Specifically, this study analyzed the relationships of CSA and associated variables (e.g., age of abuse, the sex of the perpetrator, age of perpetrator, and currently bothered by CSA) and depressive symptoms, among Black SMM. The study findings suggest that CSA experience/s, household type, the sex of the perpetrator, receiving some college education, not completing a college degree, and being unemployed and not looking for work, were positively associated with depression. Meanwhile, age of abuse, being unemployed and looking for work were negatively associated with depression. These findings suggest the need for culturally competent interventions in the Southeastern United States.

This is noteworthy since the literature and empirical research examining depression among Black SMM is scant. Black SMM typically experience multiple adverse childhood experiences such as CSA during their childhood that may predispose them to greater internalization of feelings of helplessness and hopelessness, which are consistent with depressive episode symptomology [38,49]. Further, one important issue to note here for Black SMM is that their disclosure of their CSA experiences is a process that could occur over time, which is consistent with other emerging work in this area [49,50]. Black SMM are likely to have trauma histories that may further exacerbate any trauma they experience as they progress through life, which could make them more likely to experience future trauma, especially if they continue to experience homonegative interactions with their family members, friends, or within their communities. This further increases the likelihood that they may report a depressive episode. This sample of Black SMM may also be affected by their disclosures of CSA, especially if they received anti-gay responses from the individuals that they shared this information with.

We also found that Black SMM who reported ages of abuse are more likely to report experiencing feelings of depression. This finding is consistent with some existing literature, including recovery theories from traumatic experiences such as CSA [49]. One theory of wellness and healing for adult survivors of CSA also includes disclosing and discussing the abuse with an amenable, supportive, and positive family member. An example of this is the CSA Healing Model that distinguishes between disclosure and discussing CSA [49], which is important for this population since so many in this sample reported CSA as well as feelings of depression. Having some level of social support is evident with Black SMM, since their rates of disclosing depression was high. This is notable, specifically, since social support could serve as a protective factor and may serve as a buffer against the trauma experiences they encounter with homonegative entities and people. Specifically, the ability for Black SMM to converse with supportive people who are open to these discussions about their experiences with depression will serve to strengthen their overall mental health. These findings also suggest the need to include supportive individuals in the recovery process and interventions for Black SMM with a history of CSA. Peer and social level support have proven effective in helping improve health outcomes, especially around sexual and mental health among Black SMM [51].

Other contextual factors reported by Black SMM who indicated CSA experiences were associated with their depression symptoms. Study participants who reported both male and/or female perpetrators were significantly associated with depressions symptoms. Moreover, if Black SMM were not bothered by their experience of sexual abuse, they were less likely to report symptoms of depression, possibly serving as a buffer against their depression. Black SMM could, in fact, be less bothered because they have substantial social support, from their parents, family members (of origin or chosen), or peers and may be less likely to report experiencing a depressive episode. Individuals may also have a more positive outlook, so they may be less likely to feel like they are alone even when they are experiencing adversity. As such, they may more readily internalize feelings of strength, courage, and persistence despite the adversity they may experience [52].

Lastly, our findings also indicated that Black SMM who grew up in a single mother, father, or stepparent household were more likely to report depression in comparison to those living in a two-parent household. These findings are consistent with prior literature that family structure influences youth mental health challenges [53]. Family structures have undergone significant changes in the United States over the past decades [54]. These changes in American family structure are due to same sex marriage being legal in 50 states, more adults are forgoing or delaying marriage, cohabitation on the rise, and single parents have increased [55]. Childhood family instability may severely impact Black SMM mental health due to only growing up in a single parent household but also the trauma they face for not having a second parent. In addition, to wrestling with their different identities (race, sex, gender) without the addition of the second parent figure in the household to help them with these struggles with their identities can potentially be traumatic and depressive. 

## 7. Limitations

Our study is one of the first to determine factors associated with depressive symptomology and CSA among Black SMM. However, our study is not without limitation. Black SMM and transgender women experience multiple forms of discrimination and victimization in society in addition to CSA. Therefore, we cannot attribute the existence of depression experienced by participants solely on past exposure to CSA. Our study did not examine the intersections of Black SMM and how their identities influence depression. This is important because intersectionality provides a lens in which we can examine these processes and structures that increase the risk of Black SMM experiencing CSA because of their intersecting identities. Furthermore, due to the study being a secondary data analysis, we did not assess whether participants received mental health treatment during their participation in the study, which may alter the degree in which they reported depression symptomology. Lastly, our study did not include any protective factors such as social support, which is very critical to survivors and mental health.

## 8. Practice Implications

Our results indicate the need for resources and policy change to provide culturally and safe mental health services and education on mental health in the Southeastern United States. This is importance because as a society, there is a strong need on how to discuss any kind of sexual assault, and discussion around the sexual abuse of Black SMM and men in general is rare. An investment is culturally competent mental health care providers that are located in schools, and communities that can provide services to families to teach them and their children how to respond and support their sons when they have been assaulted may help them over the course of their adulthood. When mental health providers are meeting with Black sexual minority men, they should also be aware of the intersectional stigmas that they may face and how that compounds with CSA. Providers may want to include a culturally competent CSA screening assessment, especially it may take a while for the providers and Black SMM to build trusting relationships.

## 9. Conclusions

The results have many implications for future research. First, this study demonstrated that CSA and childhood adversities have detrimental effects on depression among Black SMM. To continue to improve our understanding of how CSA impacts Black SMM survivors, future research should include how CSA impacts their intimacy and relationships; how guilt, shame and blame influences their mental and physical health and their self-esteem. In addition, how do early life stressors such as CSA affect emotional regulation and other mental health related outcomes but, also, how does CSA undermine the survivors’ ability to form trusting and meaningful relationships over the course of their life? Future research may also want to include protective factors such as social support systems. This is critical, as having a healthy social support system can potentially be a buffer for Black SMM survivors. Lastly, there is a need for more longitudinal studies on how CSA influences mental health outcomes over a Black SMM lifetime.

## Figures and Tables

**Table 1:** Sample characteristics of Black SMM in the Southeastern United States (N = 507).

Sample Characteristics	Frequency (%)
Age	
16–24	231 (43%)
25–34	160 (30%)
35–54	124 (23%)
55 and above	24 (4%)
Highest level of education	
Less than high school	38 (7%)
High school graduate or GED	174 (32%)
Some college, trade, or vocational school	213 (40%)
College graduate	78 (15%)
Graduate school	29 (5%)
More than graduate school	6 (1%)
Employment	
Working now, full-time	144 (27%)
Working now, part-time	114 (21%)
Unemployed, looking for work	216 (40%)
Unemployed, not looking for work	63 (12%)
Have other living arrangements	128 (24%)
Family Structure	
Two-Parent Household	181 (34%)
Single Mother Household	253 (48%)
Single Father Household	14 (3%)
Stepparent Household	15 (3%)
Grandparent	41 (8%)
Legal Guardian	25 (5%)

**Table 2:** Frequencies and percent of CSA variables and the outcome of depression symptoms.

Variable	Frequency	%
Depression symptoms *M (SD)*	1.69 (0.76)	0–4
Child sexual abuse (CSA) *M (SD)*	0.53	0–1
Bothered by CSA		
A lot	53	9.33
A little	88	15.49
Not at all	264	46.48
Never happened	163	28.70
Age of abuse		
1–5	40	7.62
6–10	87	16.57
11–15	120	23.00
16–18	122	23.24
Never happened	156	30.00
Age of perpetrator		
13–18	185	32.74
19–24	125	22.12
25–30	51	9.03
31-older	37	6.55
Never happened	167	29.56
Sex of perpetrator		
Male	474	89.77
Female	25	4.73
Never happened	29	5.25

Note the mean and standard deviation is reported for depression symptoms and child sexual abuse. The range for depression symptoms is 0–4, and child sexual abuse is 0–1. Child sexual abuse factors are reported as categorical.

**Table 3:** Bivariate regression with study variables on depression symptoms (N = 507).

Depression Symptoms	β	SE
Childhood sexual abuse (CSA)	0.13 **	0.08
Age of abuse (never happened reference)		
1–5 years	0.09	0.09
6–10 years	0.08	0.09
16–18	0.14 **	0.09
Do not know	0.06	0.13
Age of perpetrator (never happened reference)		
13–18 years	0.06	0.14
19–24	0.05	0.12
25–30	0.08	0.09
31–older	0.10 *	0.08
Sex of perpetrator (never happened reference)		
Male	0.22 ***	0.15
Female	0.22 ***	0.20
Family structure (two-parent household reference)		
Single mother household	0.14 **	0.07
Single father household	0.09 *	0.19
Stepparent household	0.11 *	0.20
Grandparent	0.07	0.13
Other guardian	0.08	0.15
Bothered by CSA (Never happened reference)		
Not at all	−0.09	0.08
A little	−0.10	0.09
A lot	0.13	0.09
Sex of Perpetrator (male reference)		
Female	0.22	0.53
HIV status (No, reference)	−0.10 **	0.06
Age (55 and older reference)		
35–54	0.14	0.16
25–34	0.39 ***	0.16
16–24	0.54 ***	0.15
Education (Less than HS reference)		
HS or GED	0.10	0.13
Some college, trade or vocational	0.15	0.13
College graduate	0.08	0.15
Graduate school	0.04	0.18
More than graduate School	0.04	0.33
Employment (working full-time reference)		
Working part-time	−0.05	0.09
Unemployed, looking for work	−0.11 *	0.08
Unemployed, not looking for work	0.07 *	0.11

*p* < 0.05 *, *p* < 0.01 **, *p* < 0.001 ***; Note standardized betas reported.

**Table 4:** Multiple regression with study variables on depression symptoms (N = 507).

Depression Symptoms	Beta (b)	SE
Childhood sexual abuse (CSA)	0.14 **	0.11
Age of sexual abuse (never happened reference)		
1–5 years	0.29 *	0.21
6–10 years	0.30 *	0.21
16–18	0.25 **	0.22
Do not know	0.15 *	0.22
Age of perpetrator (never happened reference)		
13–18 years	−0.21 **	0.24
19–24	−0.12	0.24
25–30	−0.20	0.24
31–older	−0.22	0.23
Sex of perpetrator (never happened reference)		
Male	0.22 ***	0.14
Female	0.19 ***	0.20
Family structure (two-parent household reference)		
Single mother household	0.10 *	0.07
Single father household	0.10 *	0.19
Stepparent household	0.10 *	0.19
Grandparent	0.05	0.12
Other guardian	0.08	0.16
Bothered by CSA (never happened reference)		
Not at all	−0.19 *	0.15
A little	−0.05	0.16
A lot	−0.02	0.16
HIV status (yes, reference)	0.03	0.07
Age (55 and older reference)		
35–54	0.11	0.16
25–34	0.35	0.17
16–24	0.50	0.17
Education (less than HS reference)		
HS or GED	−0.01	0.13
Some college, trade or vocational	0.07	0.13
College graduate	0.02	0.15
Graduate school	0.00	0.17
More than graduate school	0.04	0.31
Employment (working full time reference)		
Working part time	−0.04	0.09
Unemployed looking for work	−0.05	0.08
Unemployed not looking for work	0.03	0.11

*p* < 0.05 *, *p* < 0.01 **, *p* < 0.001 ***; Note standardized betas reported.

## Data Availability

The data is not available to the public without the consent of the PI.
